# *BAP1* tumor predisposition syndrome case report: pathological and clinical aspects of *BAP1*-inactivated melanocytic tumors (BIMTs), including dermoscopy and confocal microscopy

**DOI:** 10.1186/s12885-019-6226-8

**Published:** 2019-11-09

**Authors:** Bianca Costa Soares de Sá, Mariana Petaccia de Macedo, Giovana Tardin Torrezan, Juliana Casagrande Tavoloni Braga, Felipe Fidalgo, Luciana Facure Moredo, Rute Lellis, João Pereira Duprat, Dirce Maria Carraro

**Affiliations:** 1 Skin Cancer Department, A.C. Camargo Cancer Center, Rua Professor Antonio Prudente, 211 Liberdade, São Paulo, SP CEP: 01509-900 Brazil; 2 Department of Pathology, A.C. Camargo Cancer Center, Rua Professor Antonio Prudente, 211 Liberdade, São Paulo, SP CEP: 01509-900 Brazil; 3 Laboratory of Genomics and Molecular Biology, A.C. Camargo Cancer Center, Rua Taguá, 440, São Paulo, SP CEP: 0508-010 Brazil; 4 National Institute of Science and Technology in Oncogenomics and Therapeutic Innovation, A.C. Camargo Cancer Center, Rua Professor Antonio Prudente, 211 Liberdade, , Rua Taguá, 400, São Paulo, SP CEP: 01509-900 Brazil

**Keywords:** BIMT, BAP1, Hereditary cancer syndromes, Dermoscopy, Confocal microscopy

## Abstract

**Background:**

*BRCA1* associated-protein 1 (*BAP1*) tumor predisposition syndrome is associated with an increased risk for malignant mesotheliomas, uveal and cutaneous melanomas, renal cell carcinomas, and singular cutaneous lesions. The latter are referred to as *BAP1*-inactivated melanocytic tumors (BIMTs). When multiple BIMTs manifest, they are considered potential markers of germline *BAP1* mutations.

**Case presentation:**

Here, we report a novel pathogenic *BAP1* germline variant in a family with a history of BIMTs, cutaneous melanomas, and mesotheliomas. We also describe singular pathological aspects of the patient’s BIMT lesions and their correlation with dermoscopic and reflectance confocal microscopy findings.

**Conclusions:**

This knowledge is crucial for the recognition of BIMTs by dermatologists and pathologists, allowing the determination of appropriate management for high-risk patients, such as genetic investigations and screening for potentially aggressive tumors.

## Background

*BRCA1* associated-protein 1 (*BAP1*) tumor predisposition syndrome (*BAP1*–TPDS) is associated with the onset of cutaneous melanocytic tumors, malignant mesotheliomas, uveal and cutaneous melanomas, renal cell carcinomas, and potentially other internal malignancies [1–3].

Germline *BAP1* mutations are inherited in an autosomal dominant pattern. The main cutaneous manifestation in patients with *BAP1*–TPDS is progressive development of distinct melanocytic lesions after the first decade of life [2]. Clinically, the lesions are skin-colored to reddish-brown papules which range in diameter from 2 to 10 mm. The number of lesions vary from 5 to 50 [4]. These lesions were first reported as atypical Spitz tumors (AST), but were later considered to be a subgroup of ASTs which carry *BRAF* mutations and exhibit loss of BAP1 expression [5]. These lesions were formerly named Wiesner Nevus, BAPoma, nevoid melanoma-like melanocytic proliferations (NEMMPs) [6] or melanocytic *BAP1*-mutated atypical intradermal tumors (MBAITs) [1]. More recently, the fourth edition of the World Health Organization (WHO) Classification of Skin Tumors uses the term, *BAP1*-inactivated melanocytic tumors (BIMTs) [7]. BIMTs are estimated to occur in 75% of patients with *BAP1*–TPDS and they commonly emerge earlier than other *BAP1*-associated tumors [8]. Some authors have suggested that genetic testing for *BAP1* germline mutations should be considered for patients with two or more BIMTs [9].

To date, BIMTs have yet to be characterized by confocal microscopy, and only a few studies have described their dermoscopic aspects [8, 10, 11]. Here, we report our comprehensive characterization of the clinical and genetic traits of a *BAP1* mutation carrier. In addition, pathologic, dermoscopic, confocal, and genetic descriptions of the patient’s cutaneous tumors are reported.

## Case presentation

**Fig. 1:**
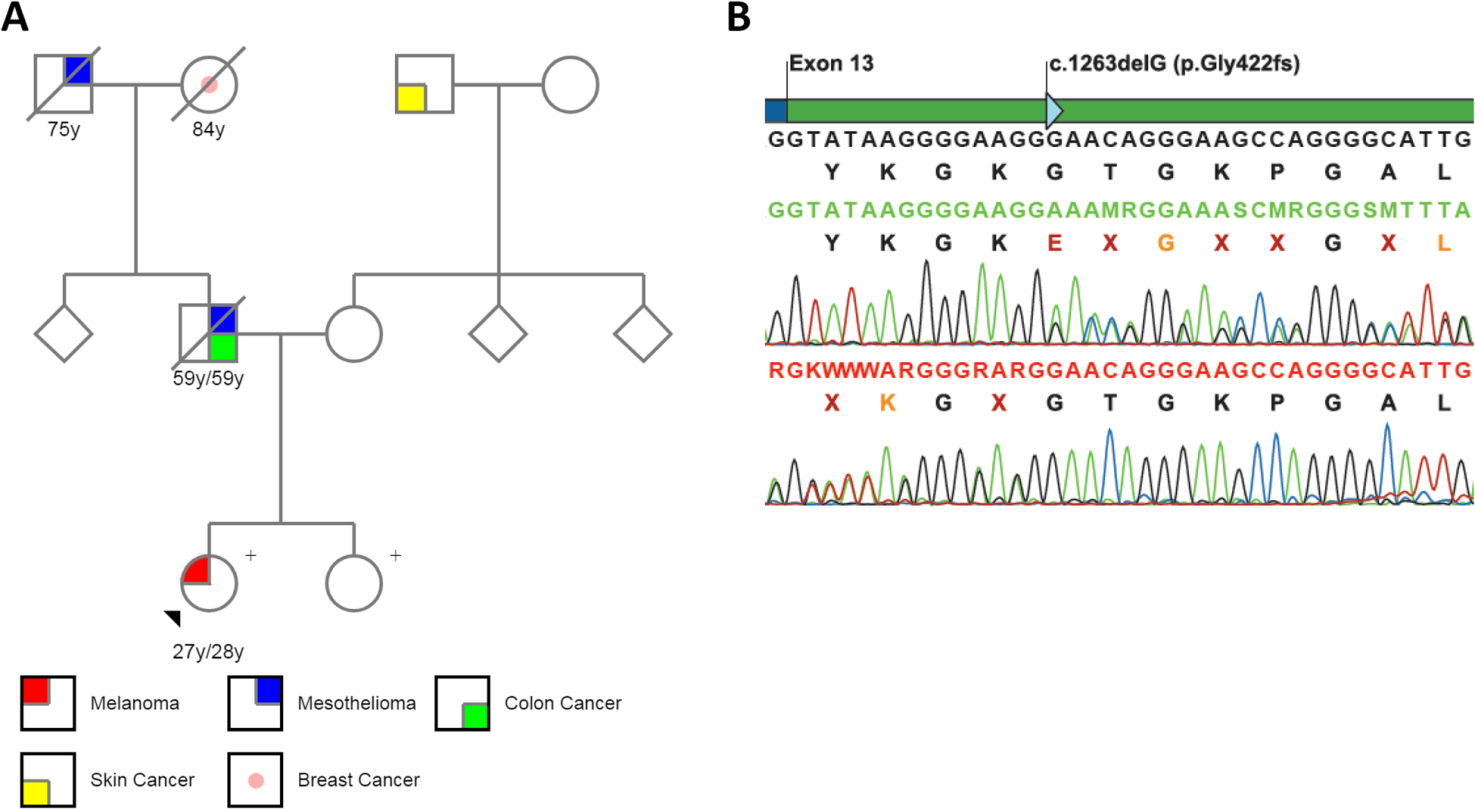
Pedigree and *BAP1* sequencing. **a** Family tree of the index case. The proband (indicated with black arrowhead) presented with cutaneous melanoma at ages 27 and 28 years, as well as with other atypical cutaneous tumors. Filled-in colored symbols indicate family members affected by cancer. When available, the age of onset for cancer is indicated underneath each individual. The two sisters (indicated with plus signs) are carriers of a *BAP1* pathogenic variant. **b** Sanger sequencing identified the c.1265delG variant (p.Gly422Glufs*8) in exon 13 of the patient’s *BAP1* gene. Sequencing chromatograms were mapped to the *BAP1* transcript reference (NM_004656) by using CLC Genomics Workbench software

A 27-year-old female was diagnosed with atypical cutaneous tumors and three melanomas. A physical examination showed Fitzpatrick type II skin, brown eyes, brown hair, and multiple melanocytic nevi, including multiple clinically intradermal nevi. The patient reported a positive history of sunburn during childhood. A detailed family history further revealed that the patient’s father was diagnosed with colon adenocarcinoma and peritoneal mesothelioma, her paternal grandfather was diagnosed with lung mesothelioma, and her paternal grandmother was diagnosed with breast cancer. The complete pedigree for the patient is represented in Fig. 1a. The patient was referred for whole body photography and digital dermoscopic follow-up of her melanocytic lesions. Genetic testing was also recommended due to her personal history of multiple melanomas and her strong family history of mesothelioma. Finally, her cutaneous tumors were submitted for hotspot mutation analysis of seven oncogenes and immunohistochemistry (IHC) to detect BAP1 expression.

### Digital Dermoscopy

A digital dermoscopy study of the patient’s melanocytic lesions was performed by two dermatologists with expertise in dermoscopy (BCCS, JCTB). FotoFinder Dermoscope® (Medicam 800 HD, TeachScreen Software, Bad Birnbach, Germany) provided a straightforward allocation and follow-up of each lesion at 20× magnification. Subsequent follow-up examinations were scheduled at intervals of 3, 6, and 12 months.

**Fig. 2:**
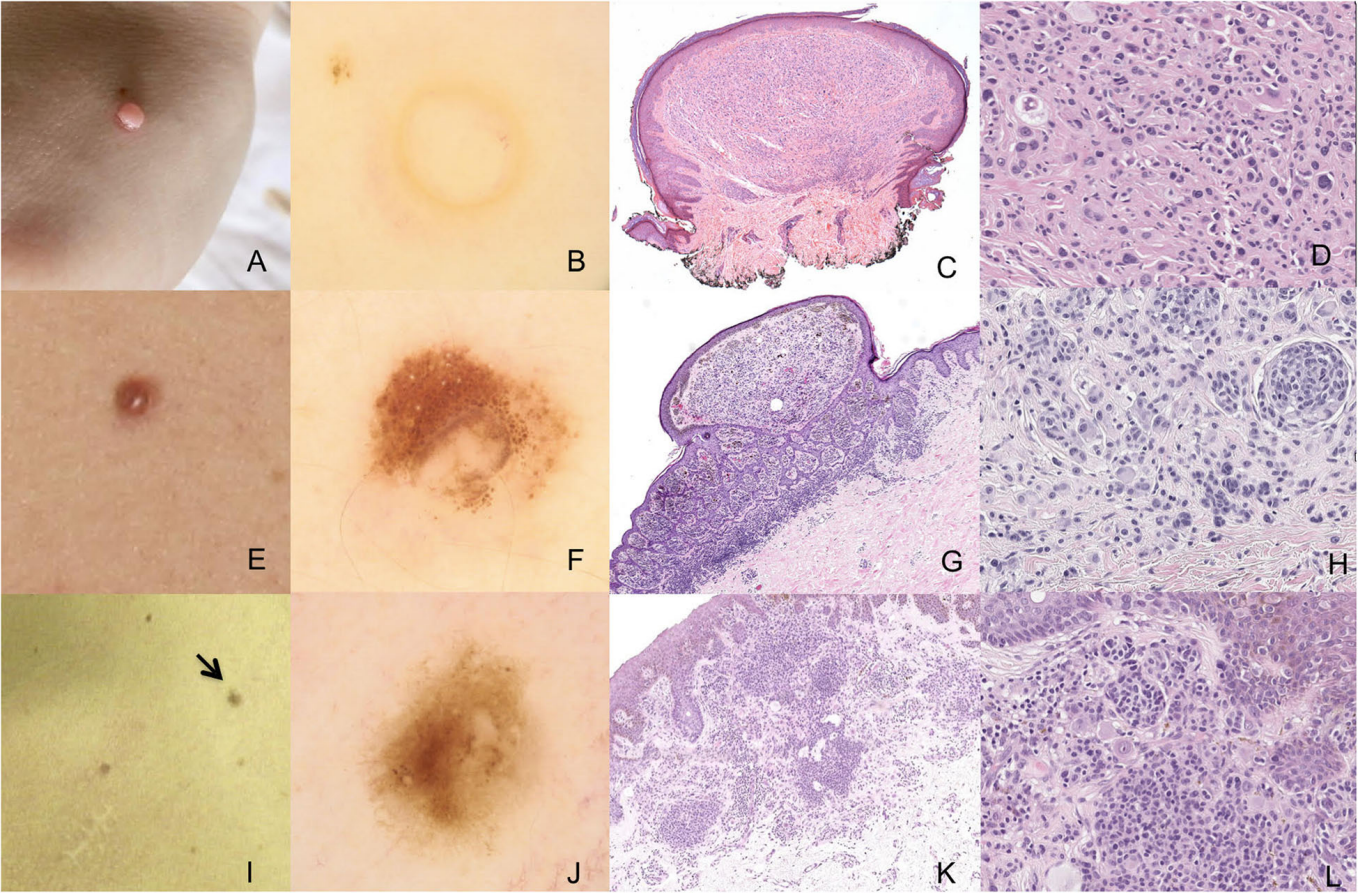
Clinical, dermoscopic, and pathologic characterizations of the skin tumors examined. For A-D, the BIMT examined was located on the back of the patient’s hand. **a** Clinical image of a skin-colored, raised tumor. **b** Dermoscopy image (20× magnification) shows a hypopigmented structureless area and discrete linear vessels at the periphery of the tumor. **c** Histology shows an intradermal, symmetrical, and well-delineated nodular melanocytic proliferation (hematoxylin & eosin (H&E), 20×) with no pigmentation. **d** At a higher magnification (200×), histology shows the lesion presents as a large, isolated group of atypical eosinophilic epithelioid cells with enlarged nuclei and abundant pink cytoplasm intermingled with smaller mature melanocytic cells (H&E). No mitosis or necrosis is observed. Clear and vacuolated cells represent adipocyte metaplasia. These findings are compatible with a diagnosis of BIMT. Loss of BAP1 expression and BRAF V600E positivity were detected in the melanocytes by IHC (data not shown). For E-H, the BIMT examined was located on the back torso of the patient. **e** Clinical image of a reddish-brown, dome-shaped papule. **f** Dermoscopy image (20× magnification) shows a central, hypopigmented structureless area surrounded by clustered brown irregular globules which vary in shape and size. **g** Histology shows a melanocytic lesion with typical junctional nests and a predominant intradermal, well-delineated nodular melanocytic proliferation. Moderate pigmentation and adipocyte metaplasia are also observed (H&E, 20× magnification). **h** At higher magnification, histology of the intradermal component (H&E, 200× magnification) shows large epithelioid cells intermingled with smaller mature melanocytic cells, compatible with a BIMT. IHC demonstrated a loss of BAP1 expression in the large cells (data not shown). Next generation sequencing additionally revealed the presence of a *BRAF* gene mutation (p.V600E). For I-L, the melanoma examined was located on the front torso of the patient. **i** Clinical image of a flat pigmented lesion (indicated with black arrow). **j** Dermoscopy image (20× magnification) shows a peripheral fine reticular network, a central brown homogenous area, irregularly distributed brown globules, and a small depigmented area. **k** Histology shows a compound, asymmetrical melanocytic lesion. The junctional component is characterized mostly by the spread of single atypical cells with upward migration, while the intradermal component includes both aggregated and diffuse cells with foci of adipocyte metaplasia (H&E, 20× magnification). **l** At higher magnification (H&E, 200×), the intradermal component is found to be composed of a large population of isolated eosinophilic epithelioid cells intermingled with smaller mature melanocytic cells. The junctional component presents a predominant lentiginous spread of large atypical epithelioid cells with pagetoid migration. The lesion is classified as an in situ melanoma associated with a background of BIMT. Sequencing further revealed this lesion as being *BRAF* wild-type

**Fig. 3:**
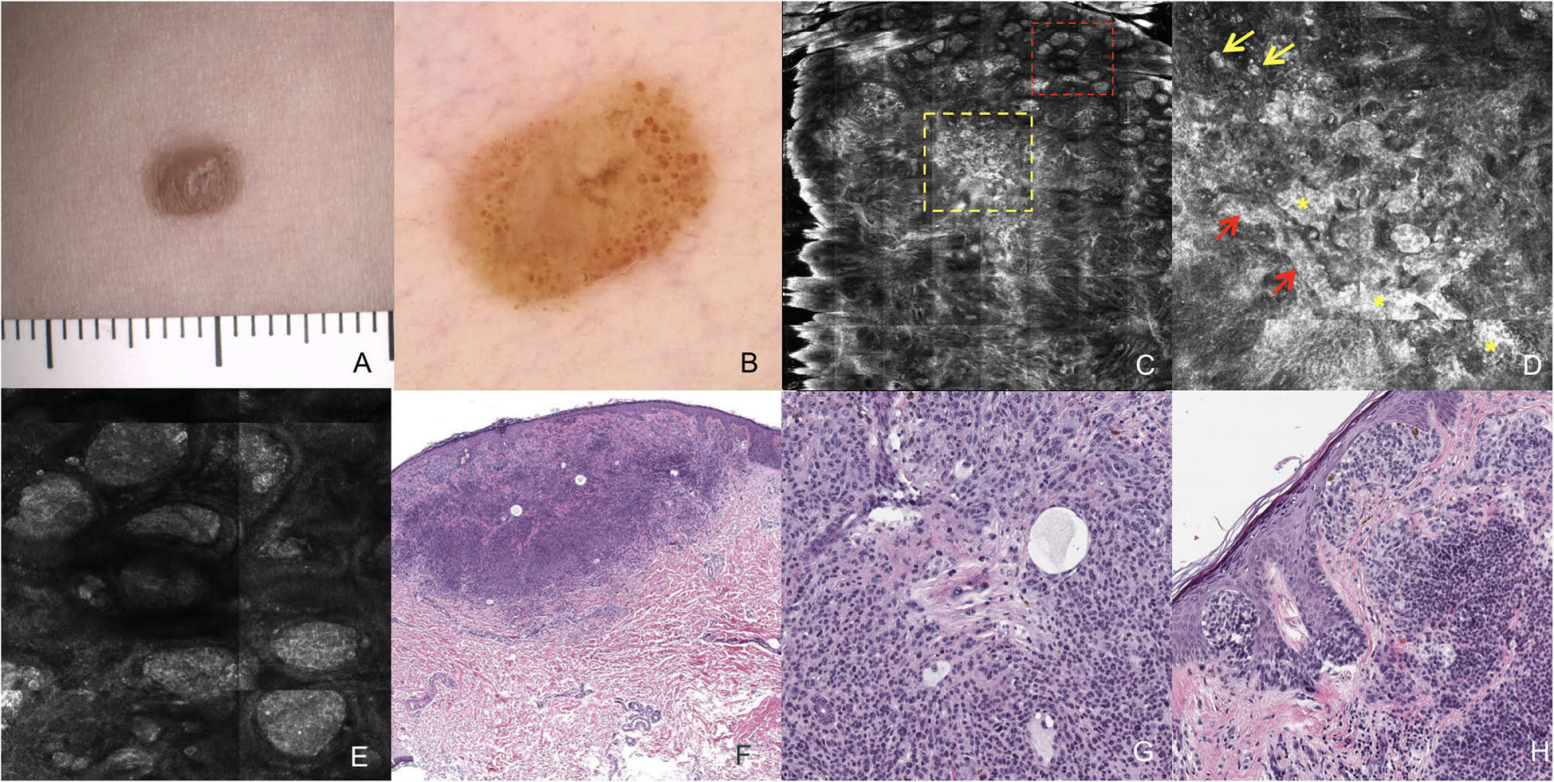
Atypical Skin Lesion – Correlations between Clinical, Dermoscopy, Pathology, and Confocal Microscopy Observations. **a** Clinical image of a brown, dome-shaped lesion. **b** Dermoscopy image (20× magnification) shows irregular pigmentation within a central light brown structureless area that is surrounded by clustered brown globules. **c** A RCM mosaic image (4 × 4 mm^2^) at the level of the DEJ shows disorganized architecture with focal loss of rete ridge meshwork. Heterogeneous brightness (marked with a yellow dashed square) and a clod pattern at the periphery (marked with a red dashed square) are also observed. **d** A RCM mosaic image (1 × 1 mm^2^) of the area inside the yellow dashed square in C at the level of the DEJ shows clusters of cells with nonhomogeneous morphologic features and reflectivity (indicated with yellow asterisks). Dendritic cells enlarged in the interpapillary spaces (indicated with red arrows) and round nucleated cells (indicated with yellow arrows) are also present. **e** An individual RCM image (0.5 × 0.5 mm^2^) of the area within the red dashed square in C at the level of the DEJ shows dense and regular nests at the periphery of the lesion. **f** Histology shows a compound, symmetrical melanocytic proliferation (H&E, 20× magnification) with benign melanocytic nests of varied sizes at the dermal-epidermal junction at the periphery of the lesion. These findings correspond to the RCM finding of a clod pattern (indicated with a red dashed square). In the center of the lesion, nest formation is reduced, corresponding to the heterogeneous brightness observed with RCM (indicated with a yellow dashed square, Fig. 3c). **g** A higher magnification (200×) image of the intradermal component (H&E) shows that the lesion includes a few isolated large epithelioid cells which are intermingled with an abundance of smaller mature melanocytic cells and foci of adipocyte metaplasia and cystic spaces. The large cells correspond to the round nucleated cells observed with RCM (indicated with yellow arrows, Fig. 3d). **h** The junctional component is composed of irregular large nests of typical melanocytes (H&E, 200× magnification). The diagnosis is compatible with BIMT. Sequencing additionally revealed this lesion harbors a *BRAF* gene mutation (p.V600E)

A total of 146 melanocytic lesions were selected for digital follow-up and all suspicious lesions were excised. A subset of the lesions were flat-pigmented and exhibited a reticular pattern by dermoscopy. There were also many dome-shaped lesions which exhibited a globular or globular-homogeneous pattern. Suspicious lesions referred for excision included those which presented peripheral, irregularly distributed brown globules and those with irregular pigmentation. Dermoscopy aspects of the BIMT lesions are detailed in Figs. 2b, f, 3b, and in Table 1.

**Table 1:** Characteristics of the BIMT lesions identified

Lesion	Clinical observations	Dermoscopic features	RCM findings	Pathology
Lesion 1 (Fig. 2a-d)	Skin-colored, dome-shaped tumor	Structureless hypopigmented area; linear vessels at periphery	–	Junctional component:
				None
				Dermal component:
				Large atypical epithelioid cells (top)
				Mature melanocytes (bottom)
				Adipocyte metaplasia (focal)
				Lack of pigmentation
				Lack of inflammation
Lesion 2 (Fig. 2e-h)	Reddish-brown papule	Central structureless, light brown area; irregular eccentric globules	–	Junctional component:
				Few nests of typical melanocytes
				Dermal component:
				Large atypical epithelioid cells (top)
				Mature melanocytes (bottom)
				Adipocyte metaplasia (focal)
				Moderate pigmentation
				Lack of inflammation
Lesion 3 (Fig. 3)	Brown papule	Clustered brown globules (periphery); irregular pigmentation within a central light brown structureless area	Dense and regular dermal nests (periphery); Sparse, isolated round nucleated cells at dermal-epidermal junction; Nonhomogeneous dermal nests (center)	Junctional component:
				Irregular large nests of typical melanocytes (periphery). Center lacking nest formation.
				Dermal component:
				Large atypical epithelioid cells (top)
				Mature melanocytes (bottom).
				Adipocyte metaplasia (focal)
				Lack of pigmentation
				Lack of inflammation

### Reflectance confocal microscopy (RCM)

RCM images were acquired with a near-infrared reflectance confocal laser scanning microscope (Vivascope 1500®; Lucid Inc., Rochester, NY, USA). Confocal image acquisition included a minimum of three mosaics (Vivablock®), each with an area of 8 × 8 mm^2^, at three different depth levels: intraepidermal, dermal-epidermal junction (DEJ), and superficial dermis. A series of high-resolution images (both capture and stack images) were also obtained at different levels from the skin surface down to the papillary dermis.

RCM was performed on two melanocytic lesions, both of which exhibited similar clinical and dermoscopic characteristics: brown dome-shaped lesions with a hypopigmented structureless area surrounded by clustered brown irregular globules which varied in shape and size. One of the lesions is shown in Fig. 3 and its RCM features are summarized in Table 1.

RCM images revealed a disorganized architecture at the center of the lesion. This architecture was characterized by an atypical honeycomb pattern in the epidermis and moderate DEJ architectural disarray (e.g., areas exhibiting partial loss of normal DEJ structure), corresponding to a central hypopigmented structureless area on dermoscopy. At the level of the DEJ, clusters of cells exhibiting nonhomogeneous morphologic features and reflectivity were observed. In addition, dendritic cells were found to enlarge the interpapillary spaces in a meshwork pattern, with isolated round nucleated cells also present (Fig. 3c and d). At the periphery, dense and regular nests of cells with similar morphologic features and reflectivity were observed (Fig. 3e). These nests corresponded with unevenly distributed brown globules observed on dermoscopy.

### Histopathology

A histopathology review of the excised lesions was performed by two dermatopathologists (MPM, RL). IHC was performed for selected lesions with a BAP1 antibody (clone C-4; 1:50 dilution, Santa Cruz Biotechnology, Dallas, TX, USA) in an automated IHC platform (Ventana BenchMark XT, Ventana Medical Systems, Tucson, AZ, USA), according to the manufacturer’s instructions.

Typical melanocytic nevi which were excised exhibited characteristics of atypical epithelioid neoplasms. Intradermal proliferation of large epithelioid melanocytes with ample eosinophilic cytoplasm and prominent nucleoli were observed. In addition, these lesions were found to be composed of different proportions of a second population of small mature-appearing melanocytic cells resembling common intradermal nevi [Fig. 2c, d, g, h, k, and l, Fig. 3f-h ]. IHC detected negative expression of BAP1 in the large epithelioid cells, while the mature-appearing melanocytes were BAP1-positive. Additional findings included focal vacuolization of cells resembling clear cells or small cystic spaces, consistent with adipocytic metaplasia [12] (Fig. 2d, g, k, Fig. 3g). Furthermore, although epithelioid cells were present, other morphologic features of Spitz Nevus, such as Kamino bodies, clefts, epidermal hyperplasia, and spindle-shaped melanocytes, were not identified.

Except for one lesion with an exclusively intradermal component (Fig. 2, C and D), the other lesions (Figs. 2g, h, and 3H) exhibited a benign junctional melanocytic component and intradermal findings typical of BIMTs. One of the lesions showed more accentuated proliferation of atypical melanocytes in the epidermis. The latter were characterized by an asymmetric distribution of epithelioid cells with large nucleoli and pronounced upward migration (Fig. 2k and l). However, despite exhibiting an intradermal BIMT component, this lesion was considered to have an associated in situ melanoma (Fig. 2k and l).

Histopathological aspects of the BIMT lesions identified are summarized in Table 1.

### Somatic mutation analysis

Genomic DNA was extracted from formalin-fixed paraffin-embedded (FFPE) tumor tissues by using a QIAamp DNA FFPE Tissue Mini Kit (Qiagen, Hilden, Germany). Targeted next generation sequencing (NGS) was subsequently performed with an Ion Proton platform and a custom Ion Ampliseq™ Panel (Thermo Fisher Scientific, Waltham, MA, USA). The latter covers hotspot regions of seven genes which are frequently mutated in solid tumors (e.g., *BRAF, EGFR, KIT, KRAS, MET, NRAS,* and *ROS1*). Mapping of sequencing reads and variant calling were performed with Torrent Suite Browser and Torrent Variant Caller (TVC) software (Thermo Fisher Scientific). Somatic mutations were defined as variant alleles present in more than 2% of reads, with a minimum coverage depth of 100 × .

Somatic mutations were investigated in six cutaneous lesions (Figs. 2 and 3). The *BRAF* V600E variant was identified in five of these lesions. However, no known hotspot oncogenic mutations were identified among the other six genes evaluated.

### Germline genetic testing

The entire coding region of *BAP1* and eight other melanoma predisposition genes (*ACD*, *CDKN2A*, *CDK4*, *MC1R*, *MITF, POT1*, *TERF2IP*, and *TERT*) were analyzed by using a custom Ion Ampliseq™ Panel (Thermo Fisher Scientific). Briefly, genomic DNA was obtained from leukocytes and then subjected to a library preparation protocol described by the Ion AmpliSeq™ Library Kit 2.0. The resulting DNA was sequenced with the Ion Proton Platform (Thermo Fisher Scientific). Variant calling files were generated by TVC 5.0–13 software and variant prioritization was performed with VarSeq software (Golden Helix, Bozeman, MT, USA). To identify rare and possibly damaging germline variants, we selected coding or splice site variants presenting coverage > 20, variant allele frequency > 30%, and minor allele frequency < 0.01 in the Exome Aggregation Consortium (ExAC) and Online Archive of Brazilian Mutations (ABraOM) databases.

In NGS-genetic testing, a heterozygous frameshift germline deletion in exon 13 was detected in the *BAP1* gene (c.1265delG; p.Gly422Glufs*8) (Fig. 1b). This deletion was not previously reported in the population databases we searched (ExAC, ABraOM, and ClinVar). Furthermore, based on phenotypic evidence and the patient’s family history of cancer, we classified the variant p.Gly422Glufs*8 as pathogenic according to recommendations of the American College of Medical Genetics (ACMG) [13]. It was further confirmed that the patient’s sister carries the same *BAP1* germline mutation, yet she had not received any prior tumor diagnosis (Fig. 1a).

## Discussion and conclusions

Here, we report a patient carrying a *BAP1* mutation who presented with multiple primary melanomas at a young age, multiple nevi, and BIMTs. In addition, two of her family members were diagnosed with mesothelioma. The comprehensive clinical, pathological, and molecular description of this case provides a valuable characterization of this rare tumor predisposing syndrome. Furthermore, the present case provides an opportunity to investigate whether dermoscopy and confocal microscopy are useful in differentiating BIMTs from other melanocytic tumors.

Recently, a multicenter study conducted by the International Dermoscopy Society described clinical and dermoscopic features of BIMTs [11]. The most frequent clinical aspect reported was pink dome-shaped papules, followed by brown papules. In the present case, three of the BIMTs examined manifested these two clinical aspects. The dermoscopic features of the present BIMT lesions also included hypopigmented structureless areas and irregular eccentric globules. This pattern was significantly more frequent among the lesions harboring a *BAP1* germline mutation, and this finding is consistent with the observations of Yelamos and collaborators [11]. However, the dermoscopic aspects of the present case differ from those of intradermal nevi which usually include a globular or globular-homogenous pattern with symmetrically distributed clustered globules and regular pigmentation [14].

RCM detected various subsurface skin features at the center of our patient’s BIMT lesions which are common to malignant melanocytic tumors (Fig. 3c and d). The features observed at the cellular level included: atypical melanocytic cells, disarrayed architecture of the DEJ, and nonhomogeneous clusters in regard to morphologic features and reflectivity. The presence of a sharp border cut-off and dense regular nests at the periphery of these lesions are findings that potentially differentiate BIMTs from melanomas [15, 16]. However, a differential diagnosis between BIMTs and melanomas may represent a diagnostic pitfall for dermatologists. Thus, additional cases need to be characterized in order to distinguish BIMTs from other melanocytic tumors with RCM.

Typically, BIMTs are microscopically described as intradermal tumors containing a dual population of large epithelioid melanocytes with cytologic atypia and pleomorphic nuclei resembling spitzoid neoplasms or rhabdoid cells [1] and a population of mature benign appearing nevoid cells. For both of these populations, mitotic activity is absent. The lesions described in the present case are consistent with these previously described characteristics of BIMTs. We also observed in the present case, as shown in previous BIMT reports [12, 17, 18], that some degree of junctional melanocytic component is associated with intradermal findings. For example, Garfield et al. [18] found that the presence of a junctional component is more common in a germline setting of BAP1 loss, rather than in a somatic setting. Thus, the new proposed WHO nomenclature of BIMT is more consistent with recent findings, with the previous nomenclature, MBAIT, drawing attention to an intradermal component. The latter could lead to a misdiagnosis by excluding lesions with junctional activity, thereby delaying screening for hereditary *BAP1*-TPDS. As described by Piris and collaborators in 2015 [17], there appears to be two histological patterns for BIMTs: a single dominant nodular pattern of epithelioid cells (Fig. 2c and d) or a dermal-nevus-like proliferation with variable numbers of epithelioid cells. Congenital onset may also be suspected if only a few of the latter nests are observed.

One of the lesions described in the present study was characterized by a striking atypical intraepidermal component with large atypical cells and pagetoid migration, consistent with a diagnosis of in situ melanoma (Fig. 2k-l). Melanomas arising in a background of a BIMT lesion are rare [12, 17]. However, the latter may indicate that BIMTs have the potential to undergo a malignant transformation. Further discussion is needed regarding the lack of pathological criteria regarding degree of junctional proliferation and/or atypia allowed in a BIMT before classifying it as an in situ melanoma.

The presence of a *BRAF* mutation in BIMT lesions is of great importance since this feature, in combination with loss of BAP1 expression, defines a distinct subset of epithelioid melanocytic tumors [4]. In only one of the lesions examined in the present study was the V600E *BRAF* mutation not detected (which was the BIMT with an in situ melanoma component). Considering that *BRAF* mutations are a common finding (90%) in BIMTs [4] and they are predicted to be maintained in tumor progression [19], we hypothesize that occasional *BRAF* negativity described in BIMTs (as demonstrated in the present case) may be due to a representation issue whereby a small proportion of large epithelioid cells is present amongst a predominance of mature-appearing cells.

The presence of vacuolated cells resembling adipocytes in BIMTs has previously been described [12, 20]. In the present study, vacuolated clear cells were observed in some of the lesions examined (Figs. 2d, k, and 3g). In the literature, these vacuolated cells have been referred to as adipocytic metaplasia. In the present study, the morphologic and IHC analyses performed demonstrate that these large cells have a vacuolated clear cell cytoplasm, a low nucleus/cytoplasm ratio, and strong positivity for Melan-A. Thus, they may correspond to clear cell melanocytes, which encompass both balloon cells and sebocyte-like cells [21]. Further analysis of clear cell melanocytes has suggested that their morphological characteristics may represent alterations in degeneration/senescence pathways which affect melanogenesis. Consequently, these melanocytes may be more likely to correspond to clear cells than adipocytic/sebocyte cells [21]. Therefore, we propose that it may be more accurate to refer to these cells as clear cells, rather than adipocytic metaplasia.

Unfortunately, we did not have access to pathology specimens from the patient’s relatives who were affected by mesothelioma to further review the subtypes present and to perform additional tests. We hypothesize that their specimens would correspond to epithelioid mesotheliomas, since these are commonly described for lesions associated with BAP1 loss [22].

In conclusion, we have reported a novel pathogenic *BAP1* germline variant present in a family affected by BIMTs, cutaneous melanomas, and mesotheliomas. In addition, we have described pathological aspects of the patient’s BIMTs and their correlation with dermoscopic findings associated with confocal features. These findings further characterize the clinical and pathological features of BIMTs, and will potentially facilitate early recognition of *BAP1* – TPDS by dermatologists and pathologists. As a result, determination of appropriate management for high-risk patients, such as genetic investigations and screenings for potentially aggressive tumors, can be achieved.

## Data Availability

All data are available within this manuscript.

## Abbreviations

ABraOM: Online Archive of Brazilian Mutations
ACMG: American College of Medical Genetics
AST: Atypical spitz tumor
BAP1: BRCA1 associated-protein 1
*BAP1*–TPDS: *BAP1* tumor predisposition syndrome
BIMT: *BAP1*-inactivated melanocytic tumor
DEJ: Dermal-epidermal junction
ExAC: Exome Aggregation Consortium
FFPE: Formalin-fixed paraffin-embedded
H&E: Hematoxylin & eosin
IHC: Immunohistochemistry
MBAIT: *BAP1*-mutated atypical intradermal tumor
NEMMP: Nevoid melanoma-like melanocytic proliferation
NGS: Next generation sequencing
RCM: Reflectance confocal microscopy
TVC: Torrent variant caller
WHO: World Health Organization
